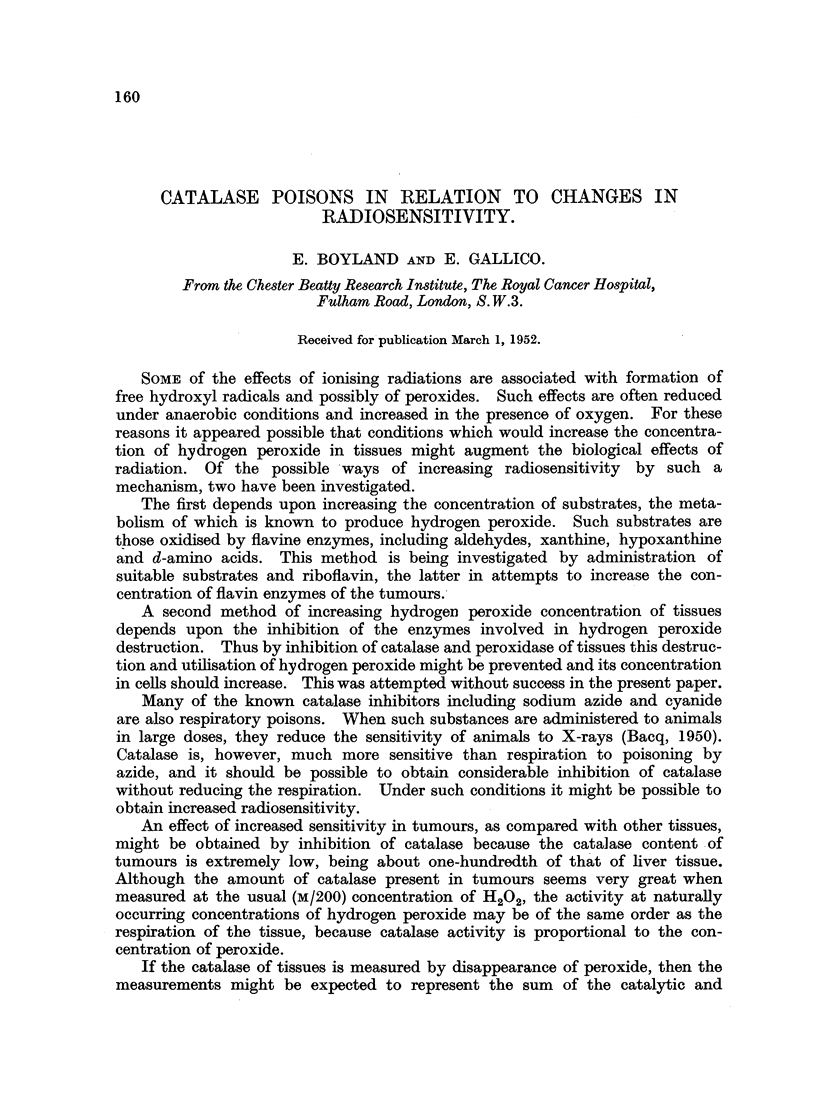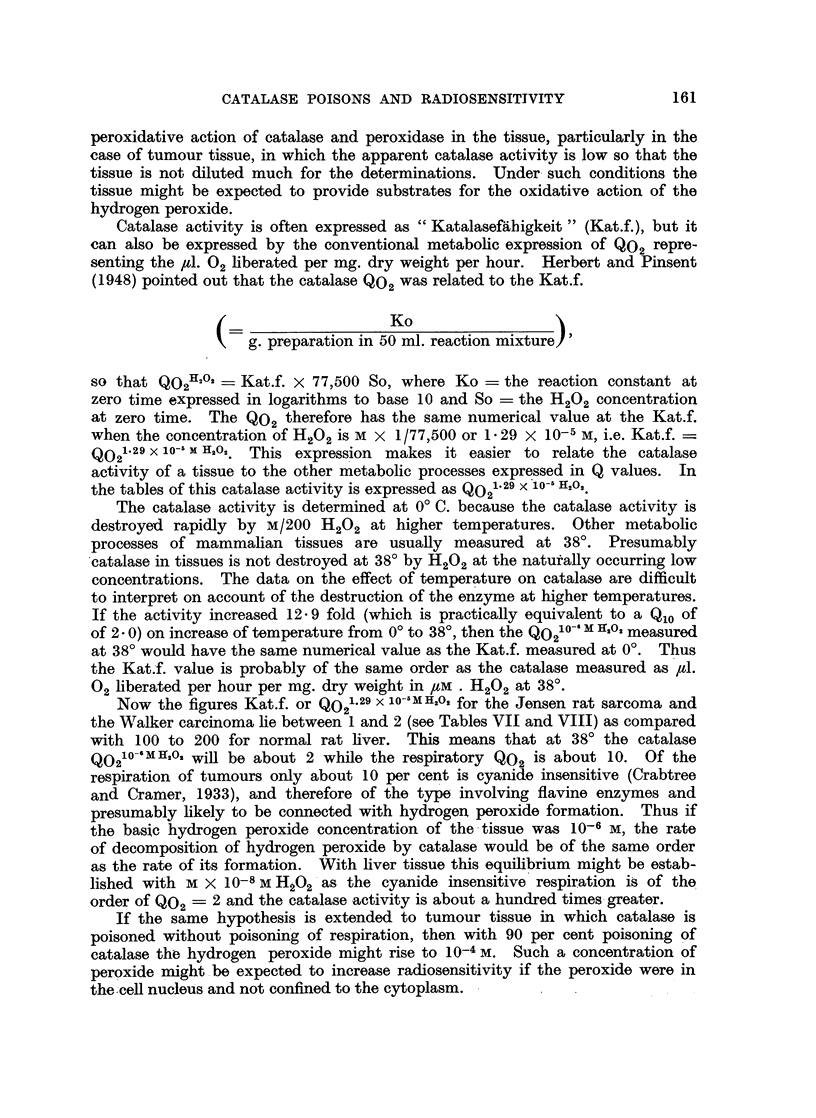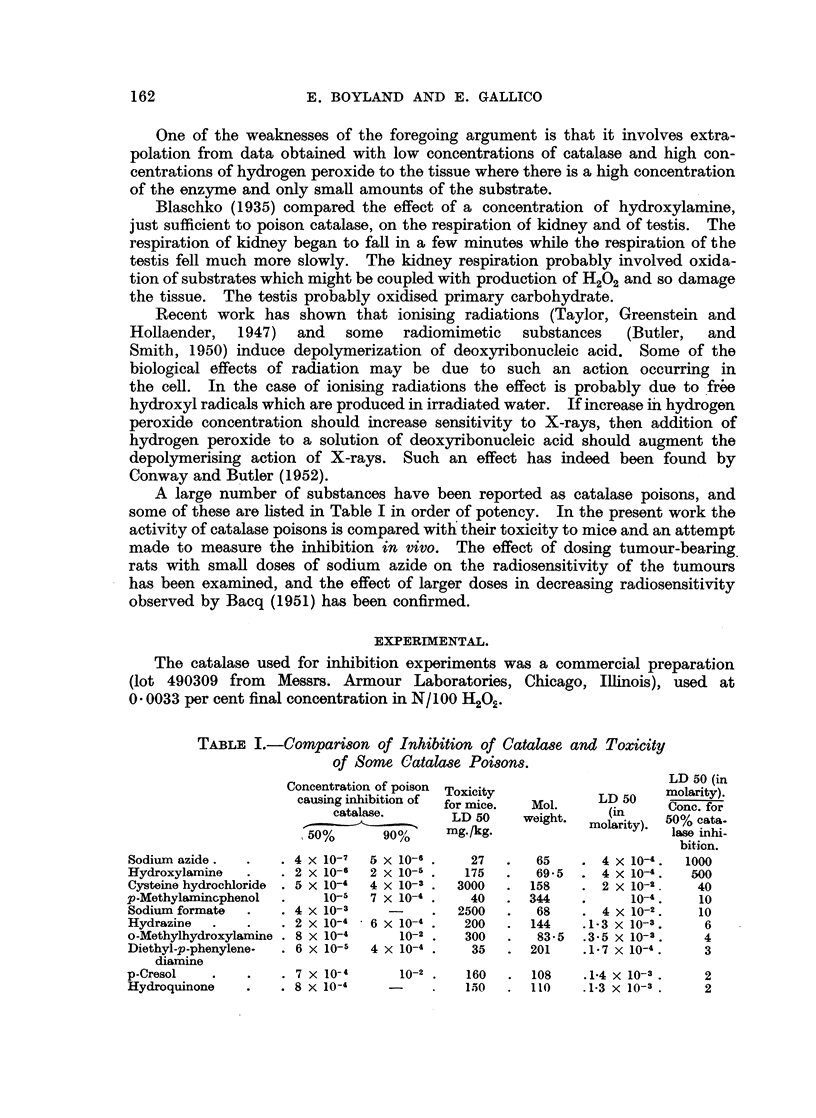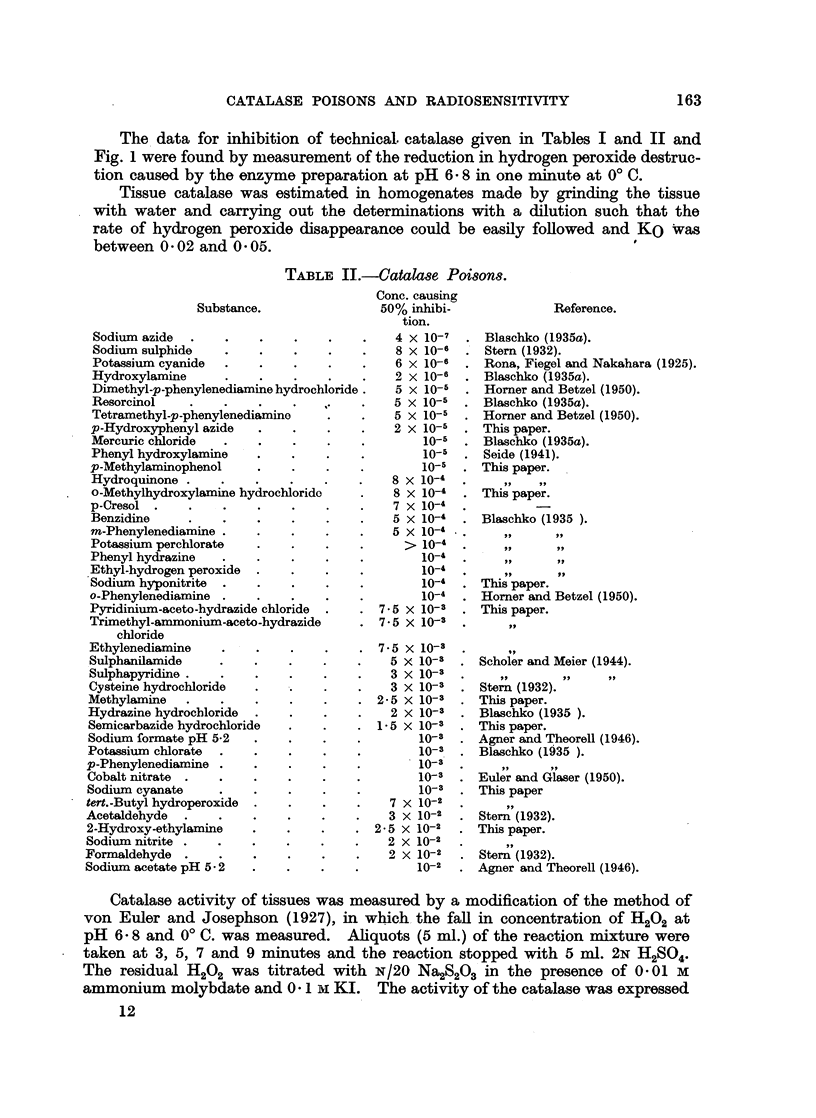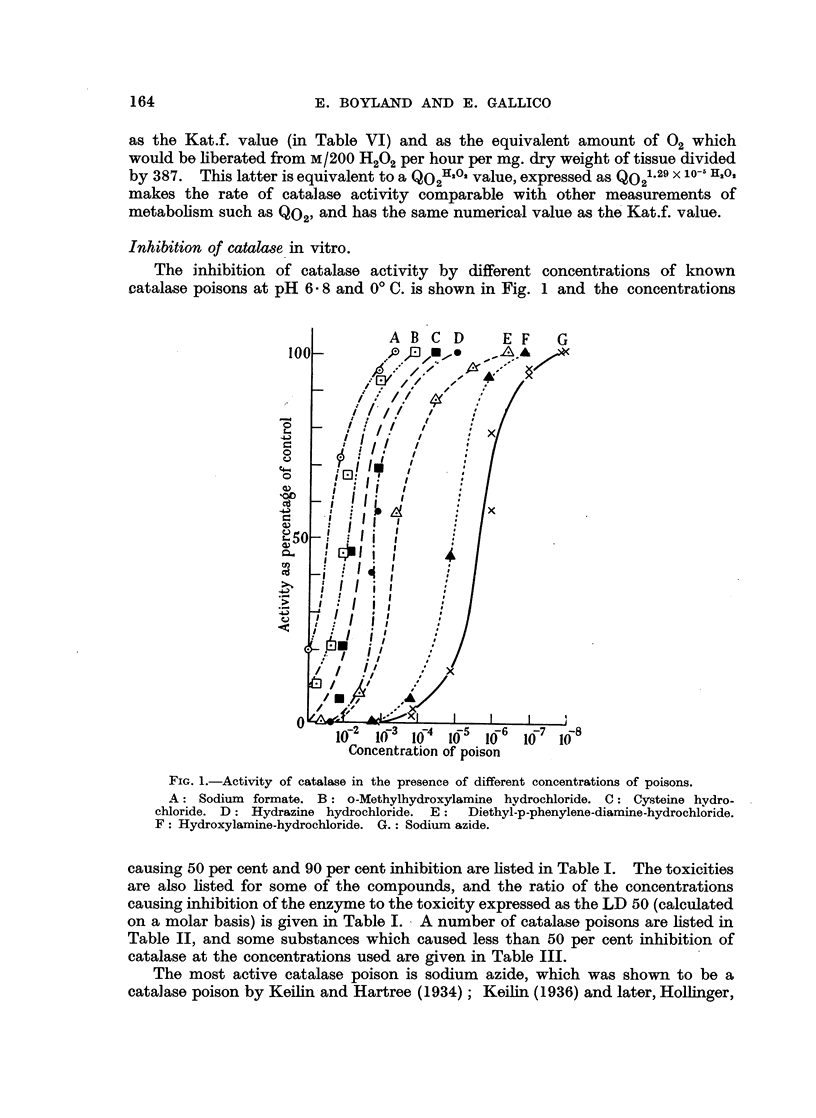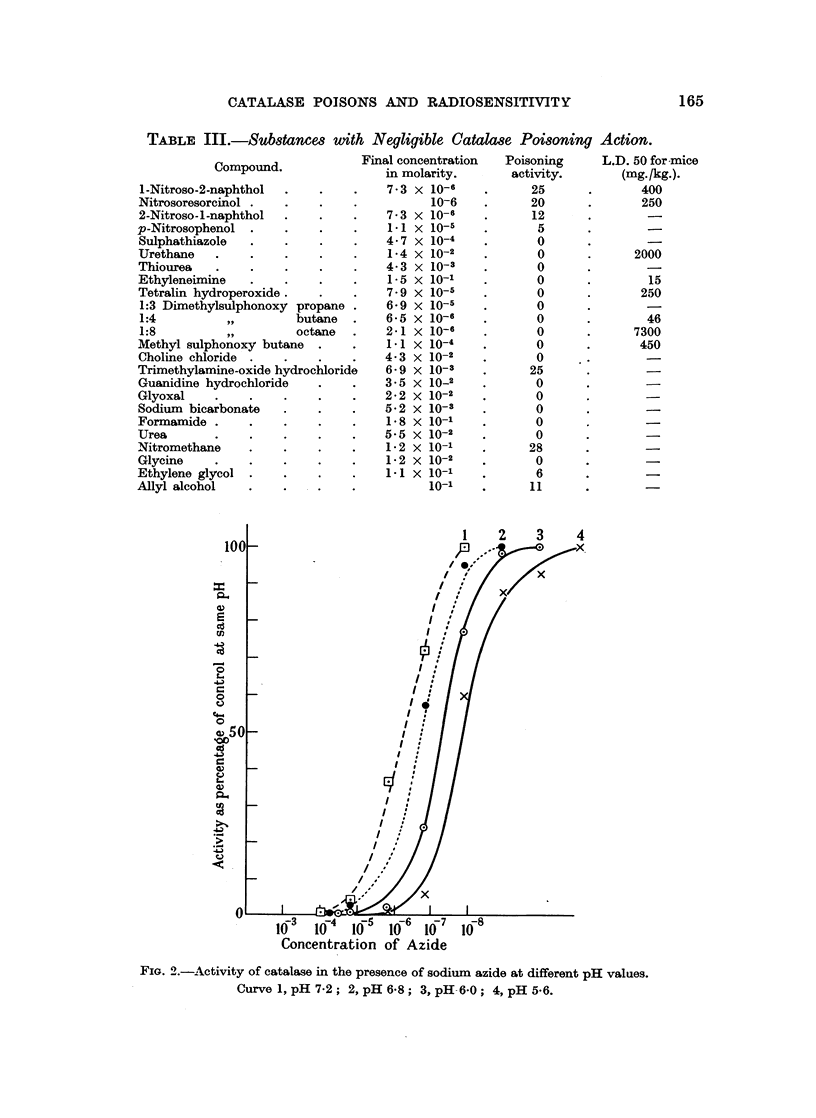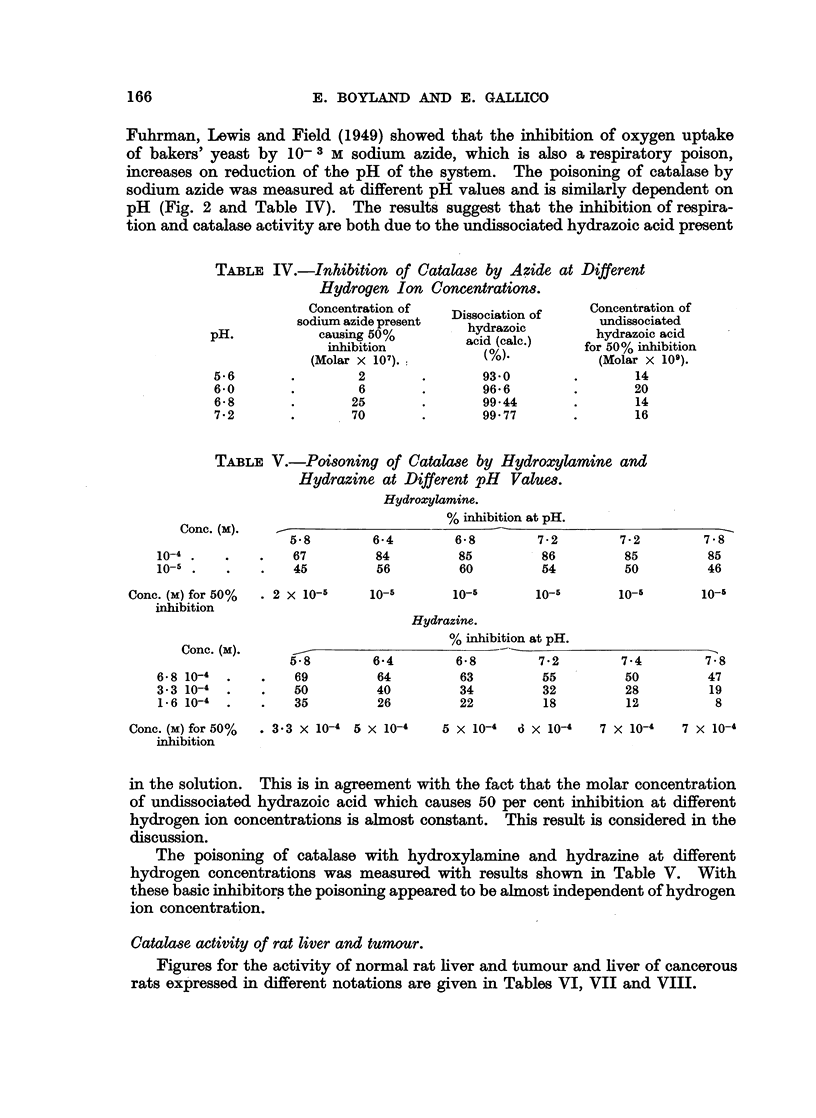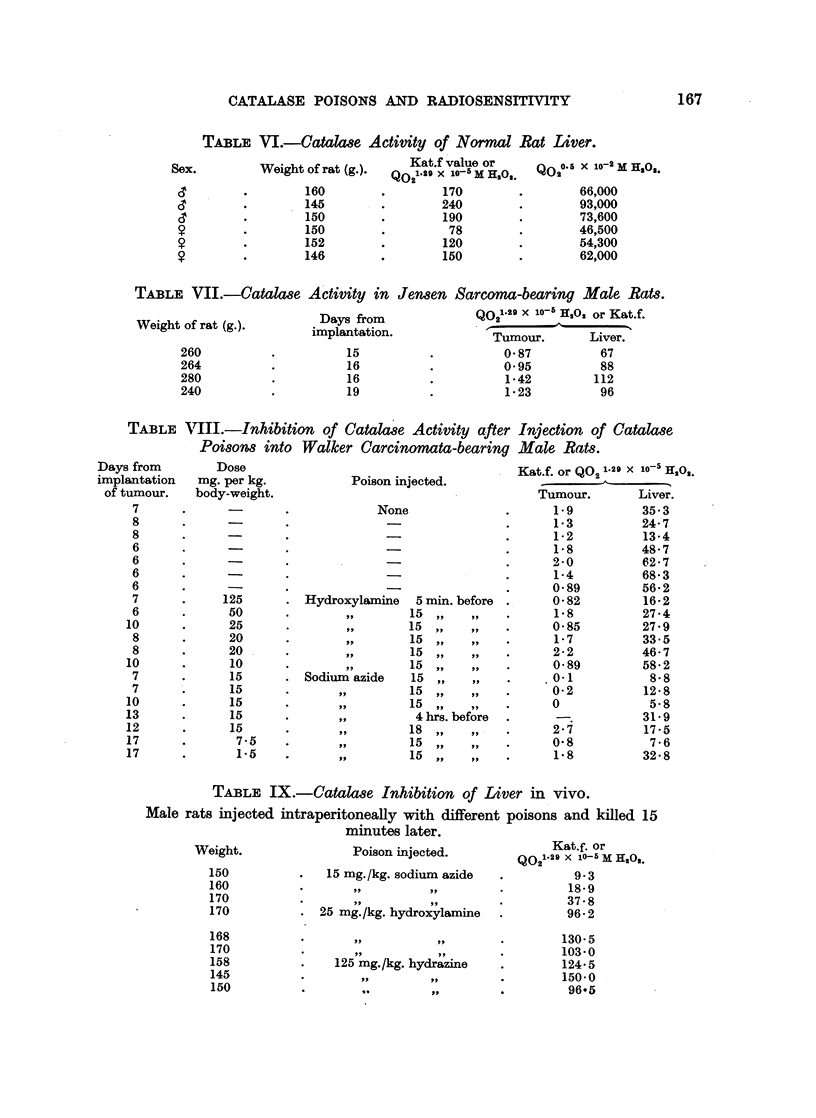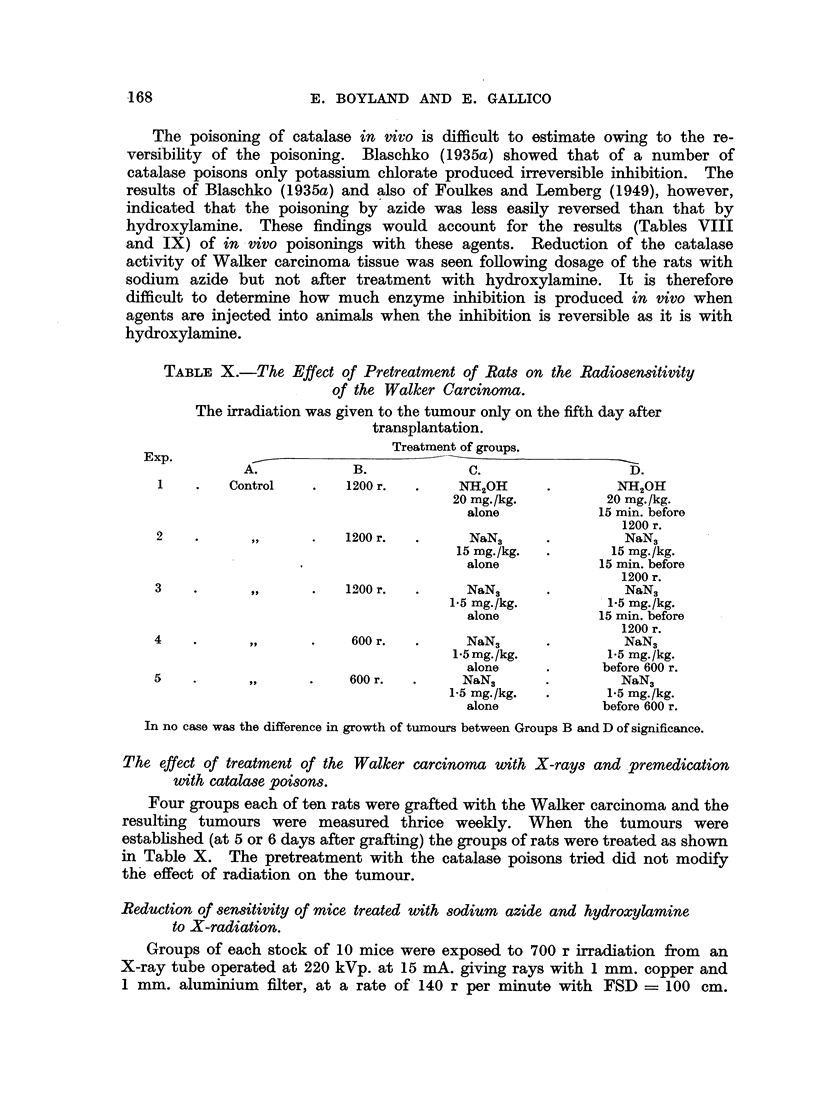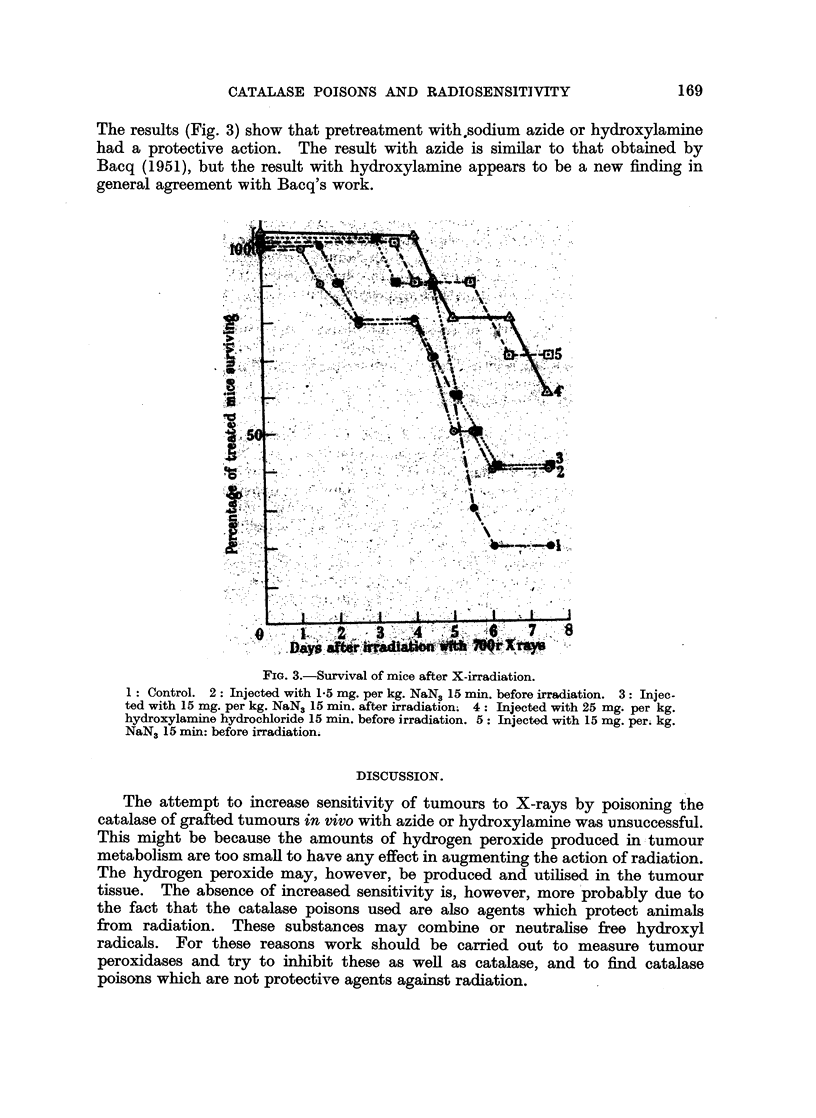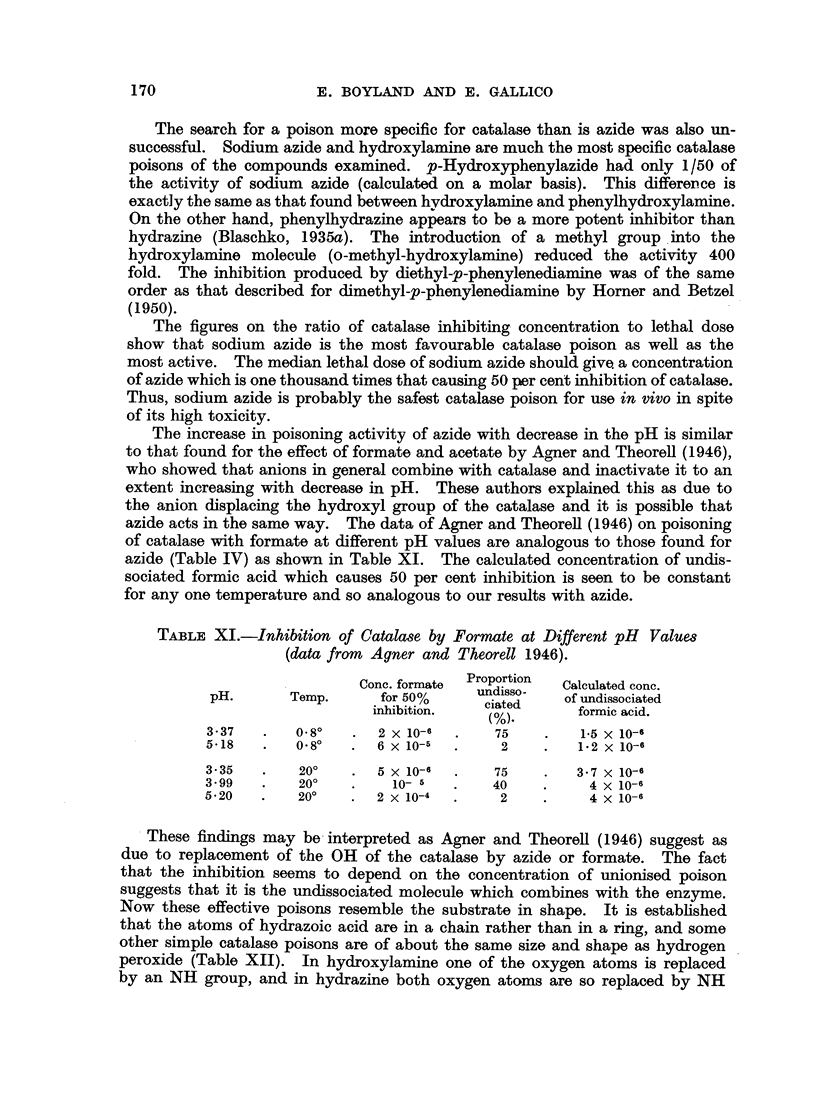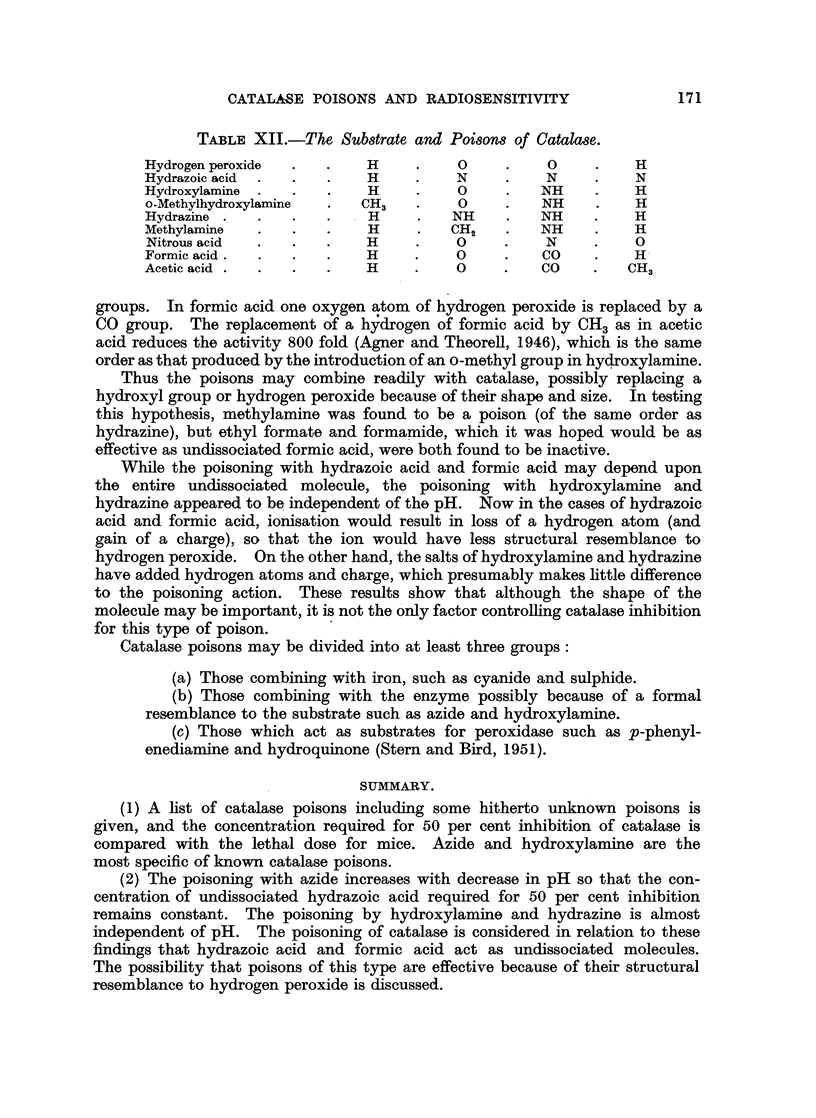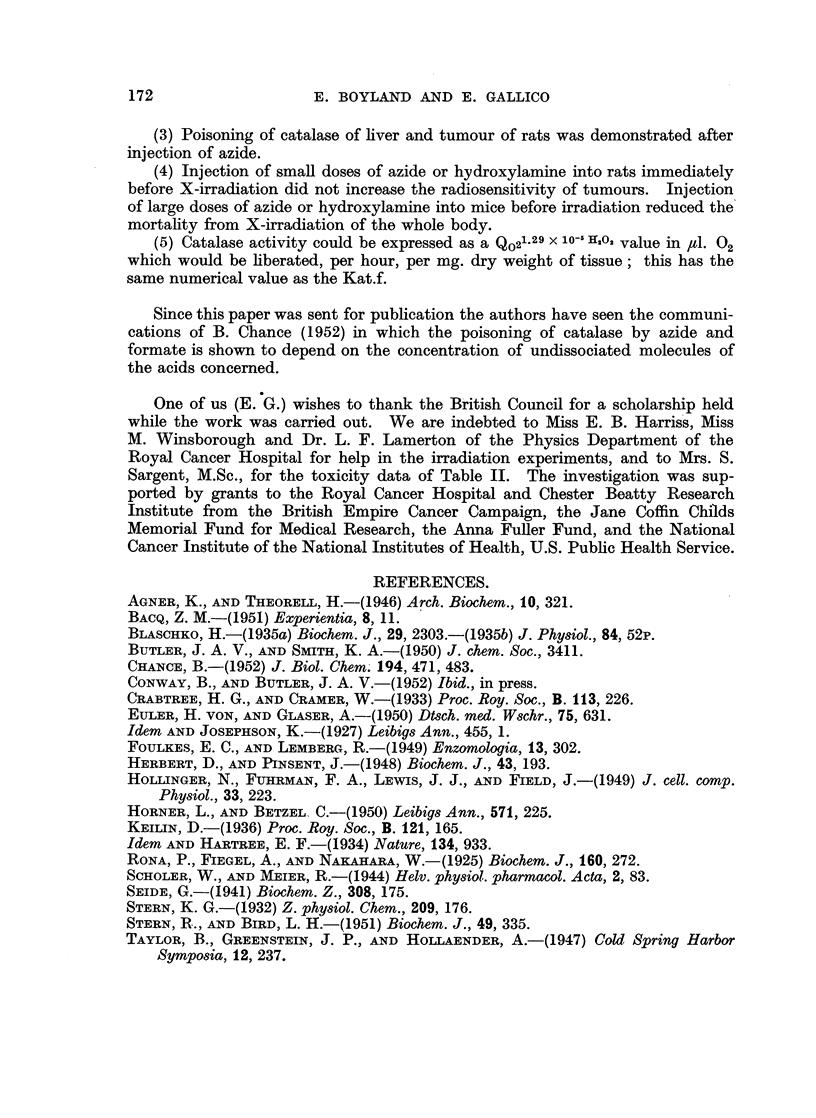# Catalase Poisons in Relation to Changes in Radiosensitivity

**DOI:** 10.1038/bjc.1952.19

**Published:** 1952-06

**Authors:** E. Boyland, E. Gallico


### CATALASE POISONS IN RELATION TO CHANGES IN RADIOSENSITIVITY.

E. BOYLAND AND E. GALLICO.

*From the Chester Beatty Research Institute, The Royal Cancer Hospital, Fulham Road, London, S.W.3.*

Received for publication March 1, 1952.

SOME of the effects of ionising radiations are associated with formation of free hydroxyl radicals and possibly of peroxides. Such effects are often reduced under anaerobic conditions and increased in the presence of oxygen. For these reasons it appeared possible that conditions which would increase the concentration of hydrogen peroxide in tissues might augment the biological effects of radiation. Of the possible ways of increasing radiosensitivity by such a mechanism, two have been investigated.

The first depends upon increasing the concentration of substrates, the metabolism of which is known to produce hydrogen peroxide. Such substrates are those oxidised by flavine enzymes, including aldehydes, xanthine, hypoxanthine and *d*-amino acids. This method is being investigated by administration of suitable substrates and riboflavin, the latter in attempts to increase the concentration of flavin enzymes of the tumours.

A second method of increasing hydrogen peroxide concentration of tissues depends upon the inhibition of the enzymes involved in hydrogen peroxide destruction. Thus by inhibition of catalase and peroxidase of tissues this destruction and utilisation of hydrogen peroxide might be prevented and its concentration in cells should increase. This was attempted without success in the present paper.

Many of the known catalase inhibitors including sodium azide and cyanide are also respiratory poisons. When such substances are administered to animals in large doses, they reduce the sensitivity of animals to X-rays (Bacq, 1950). Catalase is, however, much more sensitive than respiration to poisoning by azide, and it should be possible to obtain considerable inhibition of catalase without reducing the respiration. Under such conditions it might be possible to obtain increased radiosensitivity.

An effect of increased sensitivity in tumours, as compared with other tissues, might be obtained by inhibition of catalase because the catalase content of tumours is extremely low, being about one-hundredth of that of liver tissue. Although the amount of catalase present in tumours seems very great when measured at the usual (M/200) concentration of $H_2O_2$, the activity at naturally occurring concentrations of hydrogen peroxide may be of the same order as the respiration of the tissue, because catalase activity is proportional to the concentration of peroxide.

If the catalase of tissues is measured by disappearance of peroxide, then the measurements might be expected to represent the sum of the catalytic and peroxidative action of catalase and peroxidase in the tissue, particularly in the case of tumour tissue, in which the apparent catalase activity is low so that the tissue is not diluted much for the determinations. Under such conditions the tissue might be expected to provide substrates for the oxidative action of the hydrogen peroxide.

Catalase activity is often expressed as " Katalasefähigkeit " (Kat.f.), but it can also be expressed by the conventional metabolic expression of $Q_{O_2}$ representing the $\mu$l. $O_2$ liberated per mg. dry weight per hour. Herbert and Pinsent (1948) pointed out that the catalase $Q_{O_2}$ was related to the Kat.f.

$$\left(= \frac{\text{Ko}}{\text{g. preparation in 50 ml. reaction mixture}}\right),$$

so that $Q_{O_2}^{H_2O_2} = \text{Kat.f.} \times 77{,}500$ So, where Ko = the reaction constant at zero time expressed in logarithms to base 10 and So = the $H_2O_2$ concentration at zero time. The $Q_{O_2}$ therefore has the same numerical value at the Kat.f. when the concentration of $H_2O_2$ is M × 1/77,500 or $1 \cdot 29 \times 10^{-5}$ M, i.e. Kat.f. = $Q_{O_2}^{1 \cdot 29 \times 10^{-5} \text{ M } H_2O_2}$. This expression makes it easier to relate the catalase activity of a tissue to the other metabolic processes expressed in Q values. In the tables of this catalase activity is expressed as $Q_{O_2}^{1 \cdot 29 \times 10^{-5} H_2O_2}$.

The catalase activity is determined at 0° C. because the catalase activity is destroyed rapidly by M/200 $H_2O_2$ at higher temperatures. Other metabolic processes of mammalian tissues are usually measured at 38°. Presumably catalase in tissues is not destroyed at 38° by $H_2O_2$ at the naturally occurring low concentrations. The data on the effect of temperature on catalase are difficult to interpret on account of the destruction of the enzyme at higher temperatures. If the activity increased 12·9 fold (which is practically equivalent to a $Q_{10}$ of of 2·0) on increase of temperature from 0° to 38°, then the $Q_{O_2}^{10^{-6} \text{ M } H_2O_2}$ measured at 38° would have the same numerical value as the Kat.f. measured at 0°. Thus the Kat.f. value is probably of the same order as the catalase measured as $\mu$l. $O_2$ liberated per hour per mg. dry weight in $\mu$M . $H_2O_2$ at 38°.

Now the figures Kat.f. or $Q_{O_2}^{1.29 \times 10^{-5} \text{M} H_2O_2}$ for the Jensen rat sarcoma and the Walker carcinoma lie between 1 and 2 (see Tables VII and VIII) as compared with 100 to 200 for normal rat liver. This means that at 38° the catalase $Q_{O_2}^{10^{-6} \text{M} H_2O_2}$ will be about 2 while the respiratory $Q_{O_2}$ is about 10. Of the respiration of tumours only about 10 per cent is cyanide insensitive (Crabtree and Cramer, 1933), and therefore of the type involving flavine enzymes and presumably likely to be connected with hydrogen peroxide formation. Thus if the basic hydrogen peroxide concentration of the tissue was $10^{-6}$ M, the rate of decomposition of hydrogen peroxide by catalase would be of the same order as the rate of its formation. With liver tissue this equilibrium might be established with M × $10^{-8}$ M $H_2O_2$ as the cyanide insensitive respiration is of the order of $Q_{O_2} = 2$ and the catalase activity is about a hundred times greater.

If the same hypothesis is extended to tumour tissue in which catalase is poisoned without poisoning of respiration, then with 90 per cent poisoning of catalase the hydrogen peroxide might rise to $10^{-4}$ M. Such a concentration of peroxide might be expected to increase radiosensitivity if the peroxide were in the cell nucleus and not confined to the cytoplasm.

One of the weaknesses of the foregoing argument is that it involves extrapolation from data obtained with low concentrations of catalase and high concentrations of hydrogen peroxide to the tissue where there is a high concentration of the enzyme and only small amounts of the substrate.

Blaschko (1935) compared the effect of a concentration of hydroxylamine, just sufficient to poison catalase, on the respiration of kidney and of testis. The respiration of kidney began to fall in a few minutes while the respiration of the testis fell much more slowly. The kidney respiration probably involved oxidation of substrates which might be coupled with production of $H_2O_2$ and so damage the tissue. The testis probably oxidised primary carbohydrate.

Recent work has shown that ionising radiations (Taylor, Greenstein and Hollaender, 1947) and some radiomimetic substances (Butler, and Smith, 1950) induce depolymerization of deoxyribonucleic acid. Some of the biological effects of radiation may be due to such an action occurring in the cell. In the case of ionising radiations the effect is probably due to free hydroxyl radicals which are produced in irradiated water. If increase in hydrogen peroxide concentration should increase sensitivity to X-rays, then addition of hydrogen peroxide to a solution of deoxyribonucleic acid should augment the depolymerising action of X-rays. Such an effect has indeed been found by Conway and Butler (1952).

A large number of substances have been reported as catalase poisons, and some of these are listed in Table I in order of potency. In the present work the activity of catalase poisons is compared with their toxicity to mice and an attempt made to measure the inhibition *in vivo*. The effect of dosing tumour-bearing rats with small doses of sodium azide on the radiosensitivity of the tumours has been examined, and the effect of larger doses in decreasing radiosensitivity observed by Bacq (1951) has been confirmed.

### EXPERIMENTAL.

The catalase used for inhibition experiments was a commercial preparation (lot 490309 from Messrs. Armour Laboratories, Chicago, Illinois), used at 0·0033 per cent final concentration in N/100 $H_2O_2$.

TABLE I.—*Comparison of Inhibition of Catalase and Toxicity of Some Catalase Poisons.*

| | Concentration of poison causing inhibition of catalase. | | Toxicity for mice. LD 50 mg./kg. | Mol. weight. | LD 50 (in molarity). | LD 50 (in molarity). / Conc. for 50% catalase inhibition. |
|---|---|---|---|---|---|---|
| | 50% | 90% | | | | |
| Sodium azide | $4 \times 10^{-7}$ | $5 \times 10^{-6}$ | 27 | 65 | $4 \times 10^{-4}$ | 1000 |
| Hydroxylamine | $2 \times 10^{-6}$ | $2 \times 10^{-5}$ | 175 | 69·5 | $4 \times 10^{-4}$ | 500 |
| Cysteine hydrochloride | $5 \times 10^{-4}$ | $4 \times 10^{-3}$ | 3000 | 158 | $2 \times 10^{-2}$ | 40 |
| *p*-Methylamincphenol | $10^{-5}$ | $7 \times 10^{-4}$ | 40 | 344 | $10^{-4}$ | 10 |
| Sodium formate | $4 \times 10^{-3}$ | — | 2500 | 68 | $4 \times 10^{-2}$ | 10 |
| Hydrazine | $2 \times 10^{-4}$ | $6 \times 10^{-4}$ | 200 | 144 | $1·3 \times 10^{-3}$ | 6 |
| o-Methylhydroxylamine | $8 \times 10^{-4}$ | $10^{-2}$ | 300 | 83·5 | $3·5 \times 10^{-3}$ | 4 |
| Diethyl-*p*-phenylene-diamine | $6 \times 10^{-5}$ | $4 \times 10^{-4}$ | 35 | 201 | $1·7 \times 10^{-4}$ | 3 |
| p-Cresol | $7 \times 10^{-4}$ | $10^{-2}$ | 160 | 108 | $1·4 \times 10^{-3}$ | 2 |
| Hydroquinone | $8 \times 10^{-4}$ | — | 150 | 110 | $1·3 \times 10^{-3}$ | 2 |

The data for inhibition of technical catalase given in Tables I and II and Fig. 1 were found by measurement of the reduction in hydrogen peroxide destruction caused by the enzyme preparation at pH 6·8 in one minute at 0° C.

Tissue catalase was estimated in homogenates made by grinding the tissue with water and carrying out the determinations with a dilution such that the rate of hydrogen peroxide disappearance could be easily followed and $K_O$ was between 0·02 and 0·05.

TABLE II.—*Catalase Poisons.*

| Substance. | Conc. causing 50% inhibition. | Reference. |
|---|---|---|
| Sodium azide | $4 \times 10^{-7}$ | Blaschko (1935*a*). |
| Sodium sulphide | $8 \times 10^{-6}$ | Stern (1932). |
| Potassium cyanide | $6 \times 10^{-6}$ | Rona, Fiegel and Nakahara (1925). |
| Hydroxylamine | $2 \times 10^{-6}$ | Blaschko (1935*a*). |
| Dimethyl-*p*-phenylenediamine hydrochloride | $5 \times 10^{-5}$ | Horner and Betzel (1950). |
| Resorcinol | $5 \times 10^{-5}$ | Blaschko (1935*a*). |
| Tetramethyl-*p*-phenylenediamine | $5 \times 10^{-5}$ | Horner and Betzel (1950). |
| *p*-Hydroxyphenyl azide | $2 \times 10^{-5}$ | This paper. |
| Mercuric chloride | $10^{-5}$ | Blaschko (1935*a*). |
| Phenyl hydroxylamine | $10^{-5}$ | Seide (1941). |
| *p*-Methylaminophenol | $10^{-5}$ | This paper. |
| Hydroquinone | $8 \times 10^{-4}$ | ,, ,, |
| o-Methylhydroxylamine hydrochloride | $8 \times 10^{-4}$ | This paper. |
| p-Cresol | $7 \times 10^{-4}$ | — |
| Benzidine | $5 \times 10^{-4}$ | Blaschko (1935 ). |
| *m*-Phenylenediamine | $5 \times 10^{-4}$ | ,, ,, |
| Potassium perchlorate | $> 10^{-4}$ | ,, ,, |
| Phenyl hydrazine | $10^{-4}$ | ,, ,, |
| Ethyl-hydrogen peroxide | $10^{-4}$ | ,, ,, |
| Sodium hyponitrite | $10^{-4}$ | This paper. |
| *o*-Phenylenediamine | $10^{-4}$ | Horner and Betzel (1950). |
| Pyridinium-aceto-hydrazide chloride | $7{\cdot}5 \times 10^{-3}$ | This paper. |
| Trimethyl-ammonium-aceto-hydrazide chloride | $7{\cdot}5 \times 10^{-3}$ | ,, |
| Ethylenediamine | $7{\cdot}5 \times 10^{-3}$ | ,, |
| Sulphanilamide | $5 \times 10^{-3}$ | Scholer and Meier (1944). |
| Sulphapyridine | $3 \times 10^{-3}$ | ,, ,, ,, |
| Cysteine hydrochloride | $3 \times 10^{-3}$ | Stern (1932). |
| Methylamine | $2{\cdot}5 \times 10^{-3}$ | This paper. |
| Hydrazine hydrochloride | $2 \times 10^{-3}$ | Blaschko (1935 ). |
| Semicarbazide hydrochloride | $1{\cdot}5 \times 10^{-3}$ | This paper. |
| Sodium formate pH 5·2 | $10^{-3}$ | Agner and Theorell (1946). |
| Potassium chlorate | $10^{-3}$ | Blaschko (1935 ). |
| *p*-Phenylenediamine | $10^{-3}$ | ,, ,, |
| Cobalt nitrate | $10^{-3}$ | Euler and Glaser (1950). |
| Sodium cyanate | $10^{-3}$ | This paper |
| *tert.*-Butyl hydroperoxide | $7 \times 10^{-2}$ | ,, |
| Acetaldehyde | $3 \times 10^{-2}$ | Stern (1932). |
| 2-Hydroxy-ethylamine | $2{\cdot}5 \times 10^{-2}$ | This paper. |
| Sodium nitrite | $2 \times 10^{-2}$ | ,, |
| Formaldehyde | $2 \times 10^{-2}$ | Stern (1932). |
| Sodium acetate pH 5·2 | $10^{-2}$ | Agner and Theorell (1946). |

Catalase activity of tissues was measured by a modification of the method of von Euler and Josephson (1927), in which the fall in concentration of $H_2O_2$ at pH 6·8 and 0° C. was measured. Aliquots (5 ml.) of the reaction mixture were taken at 3, 5, 7 and 9 minutes and the reaction stopped with 5 ml. 2N $H_2SO_4$. The residual $H_2O_2$ was titrated with N/20 $Na_2S_2O_3$ in the presence of 0·01 M ammonium molybdate and 0·1 M KI. The activity of the catalase was expressed as the Kat.f. value (in Table VI) and as the equivalent amount of $O_2$ which would be liberated from M/200 $H_2O_2$ per hour per mg. dry weight of tissue divided by 387. This latter is equivalent to a $QO_2^{H_2O_2}$ value, expressed as $QO_2^{1.29\times10^{-5}\,H_2O_2}$ makes the rate of catalase activity comparable with other measurements of metabolism such as $QO_2$, and has the same numerical value as the Kat.f. value.

*Inhibition of catalase* in vitro.

The inhibition of catalase activity by different concentrations of known catalase poisons at pH 6·8 and 0° C. is shown in Fig. 1 and the concentrations

FIG. 1.—Activity of catalase in the presence of different concentrations of poisons.
A: Sodium formate. B: o-Methylhydroxylamine hydrochloride. C: Cysteine hydrochloride. D: Hydrazine hydrochloride. E: Diethyl-p-phenylene-diamine-hydrochloride. F: Hydroxylamine-hydrochloride. G.: Sodium azide.

causing 50 per cent and 90 per cent inhibition are listed in Table I. The toxicities are also listed for some of the compounds, and the ratio of the concentrations causing inhibition of the enzyme to the toxicity expressed as the LD 50 (calculated on a molar basis) is given in Table I. A number of catalase poisons are listed in Table II, and some substances which caused less than 50 per cent inhibition of catalase at the concentrations used are given in Table III.

The most active catalase poison is sodium azide, which was shown to be a catalase poison by Keilin and Hartree (1934); Keilin (1936) and later, Hollinger,

TABLE III.—*Substances with Negligible Catalase Poisoning Action.*

| Compound. | Final concentration in molarity. | Poisoning activity. | L.D. 50 for mice (mg./kg.). |
|---|---|---|---|
| 1-Nitroso-2-naphthol | $7 \cdot 3 \times 10^{-6}$ | 25 | 400 |
| Nitrosoresorcinol | $10^{-6}$ | 20 | 250 |
| 2-Nitroso-1-naphthol | $7 \cdot 3 \times 10^{-6}$ | 12 | — |
| *p*-Nitrosophenol | $1 \cdot 1 \times 10^{-5}$ | 5 | — |
| Sulphathiazole | $4 \cdot 7 \times 10^{-4}$ | 0 | — |
| Urethane | $1 \cdot 4 \times 10^{-2}$ | 0 | 2000 |
| Thiourea | $4 \cdot 3 \times 10^{-3}$ | 0 | — |
| Ethyleneimine | $1 \cdot 5 \times 10^{-1}$ | 0 | 15 |
| Tetralin hydroperoxide | $7 \cdot 9 \times 10^{-5}$ | 0 | 250 |
| 1:3 Dimethylsulphonoxy propane | $6 \cdot 9 \times 10^{-5}$ | 0 | — |
| 1:4 „ butane | $6 \cdot 5 \times 10^{-6}$ | 0 | 46 |
| 1:8 „ octane | $2 \cdot 1 \times 10^{-6}$ | 0 | 7300 |
| Methyl sulphonoxy butane | $1 \cdot 1 \times 10^{-4}$ | 0 | 450 |
| Choline chloride | $4 \cdot 3 \times 10^{-2}$ | 0 | — |
| Trimethylamine-oxide hydrochloride | $6 \cdot 9 \times 10^{-3}$ | 25 | — |
| Guanidine hydrochloride | $3 \cdot 5 \times 10^{-2}$ | 0 | — |
| Glyoxal | $2 \cdot 2 \times 10^{-2}$ | 0 | — |
| Sodium bicarbonate | $5 \cdot 2 \times 10^{-3}$ | 0 | — |
| Formamide | $1 \cdot 8 \times 10^{-1}$ | 0 | — |
| Urea | $5 \cdot 5 \times 10^{-2}$ | 0 | — |
| Nitromethane | $1 \cdot 2 \times 10^{-1}$ | 28 | — |
| Glycine | $1 \cdot 2 \times 10^{-2}$ | 0 | — |
| Ethylene glycol | $1 \cdot 1 \times 10^{-1}$ | 6 | — |
| Allyl alcohol | $10^{-1}$ | 11 | — |

FIG. 2.—Activity of catalase in the presence of sodium azide at different pH values. Curve 1, pH 7·2 ; 2, pH 6·8 ; 3, pH 6·0 ; 4, pH 5·6.

Fuhrman, Lewis and Field (1949) showed that the inhibition of oxygen uptake of bakers' yeast by $10^{-3}$ M sodium azide, which is also a respiratory poison, increases on reduction of the pH of the system. The poisoning of catalase by sodium azide was measured at different pH values and is similarly dependent on pH (Fig. 2 and Table IV). The results suggest that the inhibition of respiration and catalase activity are both due to the undissociated hydrazoic acid present

TABLE IV.—*Inhibition of Catalase by Azide at Different Hydrogen Ion Concentrations.*

| pH. | Concentration of sodium azide present causing 50% inhibition (Molar $\times 10^7$). | Dissociation of hydrazoic acid (calc.) (%). | Concentration of undissociated hydrazoic acid for 50% inhibition (Molar $\times 10^9$). |
|---|---|---|---|
| 5·6 | 2 | 93·0 | 14 |
| 6·0 | 6 | 96·6 | 20 |
| 6·8 | 25 | 99·44 | 14 |
| 7·2 | 70 | 99·77 | 16 |

TABLE V.—*Poisoning of Catalase by Hydroxylamine and Hydrazine at Different pH Values.*

*Hydroxylamine.*

| Conc. (M). | % inhibition at pH. | | | | | |
|---|---|---|---|---|---|---|
| | 5·8 | 6·4 | 6·8 | 7·2 | 7·2 | 7·8 |
| $10^{-4}$ | 67 | 84 | 85 | 86 | 85 | 85 |
| $10^{-5}$ | 45 | 56 | 60 | 54 | 50 | 46 |
| Conc. (M) for 50% inhibition | $2 \times 10^{-5}$ | $10^{-5}$ | $10^{-5}$ | $10^{-5}$ | $10^{-5}$ | $10^{-5}$ |

*Hydrazine.*

| Conc. (M). | % inhibition at pH. | | | | | |
|---|---|---|---|---|---|---|
| | 5·8 | 6·4 | 6·8 | 7·2 | 7·4 | 7·8 |
| $6·8\ 10^{-4}$ | 69 | 64 | 63 | 55 | 50 | 47 |
| $3·3\ 10^{-4}$ | 50 | 40 | 34 | 32 | 28 | 19 |
| $1·6\ 10^{-4}$ | 35 | 26 | 22 | 18 | 12 | 8 |
| Conc. (M) for 50% inhibition | $3·3 \times 10^{-4}$ | $5 \times 10^{-4}$ | $5 \times 10^{-4}$ | $6 \times 10^{-4}$ | $7 \times 10^{-4}$ | $7 \times 10^{-4}$ |

in the solution. This is in agreement with the fact that the molar concentration of undissociated hydrazoic acid which causes 50 per cent inhibition at different hydrogen ion concentrations is almost constant. This result is considered in the discussion.

The poisoning of catalase with hydroxylamine and hydrazine at different hydrogen concentrations was measured with results shown in Table V. With these basic inhibitors the poisoning appeared to be almost independent of hydrogen ion concentration.

*Catalase activity of rat liver and tumour.*

Figures for the activity of normal rat liver and tumour and liver of cancerous rats expressed in different notations are given in Tables VI, VII and VIII.

TABLE VI.—*Catalase Activity of Normal Rat Liver.*

| Sex. | Weight of rat (g.). | Kat.f value or $QO_2^{1.29} \times 10^{-5}$ M $H_2O_2$. | $QO_2^{0.5} \times 10^{-2}$ M $H_2O_2$. |
|---|---|---|---|
| ♂ | 160 | 170 | 66,000 |
| ♂ | 145 | 240 | 93,000 |
| ♂ | 150 | 190 | 73,600 |
| ♀ | 150 | 78 | 46,500 |
| ♀ | 152 | 120 | 54,300 |
| ♀ | 146 | 150 | 62,000 |

TABLE VII.—*Catalase Activity in Jensen Sarcoma-bearing Male Rats.*

| Weight of rat (g.). | Days from implantation. | $QO_2^{1.29} \times 10^{-5}$ $H_2O_2$ or Kat.f. | |
|---|---|---|---|
| | | Tumour. | Liver. |
| 260 | 15 | 0·87 | 67 |
| 264 | 16 | 0·95 | 88 |
| 280 | 16 | 1·42 | 112 |
| 240 | 19 | 1·23 | 96 |

TABLE VIII.—*Inhibition of Catalase Activity after Injection of Catalase Poisons into Walker Carcinomata-bearing Male Rats.*

| Days from implantation of tumour. | Dose mg. per kg. body-weight. | Poison injected. | | Kat.f. or $QO_2^{1.29} \times 10^{-5}$ $H_2O_2$. | |
|---|---|---|---|---|---|
| | | | | Tumour. | Liver. |
| 7 | — | None | | 1·9 | 35·3 |
| 8 | — | — | | 1·3 | 24·7 |
| 8 | — | — | | 1·2 | 13·4 |
| 6 | — | — | | 1·8 | 48·7 |
| 6 | — | — | | 2·0 | 62·7 |
| 6 | — | — | | 1·4 | 68·3 |
| 6 | — | — | | 0·89 | 56·2 |
| 7 | 125 | Hydroxylamine | 5 min. before | 0·82 | 16·2 |
| 6 | 50 | ,, | 15 ,, ,, | 1·8 | 27·4 |
| 10 | 25 | ,, | 15 ,, ,, | 0·85 | 27·9 |
| 8 | 20 | ,, | 15 ,, ,, | 1·7 | 33·5 |
| 8 | 20 | ,, | 15 ,, ,, | 2·2 | 46·7 |
| 10 | 10 | ,, | 15 ,, ,, | 0·89 | 58·2 |
| 7 | 15 | Sodium azide | 15 ,, ,, | 0·1 | 8·8 |
| 7 | 15 | ,, | 15 ,, ,, | 0·2 | 12·8 |
| 10 | 15 | ,, | 15 ,, ,, | 0 | 5·8 |
| 13 | 15 | ,, | 4 hrs. before | — | 31·9 |
| 12 | 15 | ,, | 18 ,, ,, | 2·7 | 17·5 |
| 17 | 7·5 | ,, | 15 ,, ,, | 0·8 | 7·6 |
| 17 | 1·5 | ,, | 15 ,, ,, | 1·8 | 32·8 |

TABLE IX.—*Catalase Inhibition of Liver* in vivo.

Male rats injected intraperitoneally with different poisons and killed 15 minutes later.

| Weight. | Poison injected. | Kat.f. or $QO_2^{1.29} \times 10^{-5}$ M $H_2O_2$. |
|---|---|---|
| 150 | 15 mg./kg. sodium azide | 9·3 |
| 160 | ,, ,, | 18·9 |
| 170 | ,, ,, | 37·8 |
| 170 | 25 mg./kg. hydroxylamine | 96·2 |
| 168 | ,, ,, | 130·5 |
| 170 | ,, ,, | 103·0 |
| 158 | 125 mg./kg. hydrazine | 124·5 |
| 145 | ,, ,, | 150·0 |
| 150 | ,, ,, | 96·5 |

The poisoning of catalase *in vivo* is difficult to estimate owing to the reversibility of the poisoning. Blaschko (1935*a*) showed that of a number of catalase poisons only potassium chlorate produced irreversible inhibition. The results of Blaschko (1935*a*) and also of Foulkes and Lemberg (1949), however, indicated that the poisoning by azide was less easily reversed than that by hydroxylamine. These findings would account for the results (Tables VIII and IX) of *in vivo* poisonings with these agents. Reduction of the catalase activity of Walker carcinoma tissue was seen following dosage of the rats with sodium azide but not after treatment with hydroxylamine. It is therefore difficult to determine how much enzyme inhibition is produced *in vivo* when agents are injected into animals when the inhibition is reversible as it is with hydroxylamine.

TABLE X.—*The Effect of Pretreatment of Rats on the Radiosensitivity of the Walker Carcinoma.*

The irradiation was given to the tumour only on the fifth day after transplantation.

| Exp. | Treatment of groups. A. | B. | C. | D. |
|---|---|---|---|---|
| 1 | Control | 1200 r. | $NH_2OH$ 20 mg./kg. alone | $NH_2OH$ 20 mg./kg. 15 min. before 1200 r. |
| 2 | ,, | 1200 r. | $NaN_3$ 15 mg./kg. alone | $NaN_3$ 15 mg./kg. 15 min. before 1200 r. |
| 3 | ,, | 1200 r. | $NaN_3$ 1·5 mg./kg. alone | $NaN_3$ 1·5 mg./kg. 15 min. before 1200 r. |
| 4 | ,, | 600 r. | $NaN_3$ 1·5 mg./kg. alone | $NaN_3$ 1·5 mg./kg. before 600 r. |
| 5 | ,, | 600 r. | $NaN_3$ 1·5 mg./kg. alone | $NaN_3$ 1·5 mg./kg. before 600 r. |

In no case was the difference in growth of tumours between Groups B and D of significance.

*The effect of treatment of the Walker carcinoma with X-rays and premedication with catalase poisons.*

Four groups each of ten rats were grafted with the Walker carcinoma and the resulting tumours were measured thrice weekly. When the tumours were established (at 5 or 6 days after grafting) the groups of rats were treated as shown in Table X. The pretreatment with the catalase poisons tried did not modify the effect of radiation on the tumour.

*Reduction of sensitivity of mice treated with sodium azide and hydroxylamine to X-radiation.*

Groups of each stock of 10 mice were exposed to 700 r irradiation from an X-ray tube operated at 220 kVp. at 15 mA. giving rays with 1 mm. copper and 1 mm. aluminium filter, at a rate of 140 r per minute with FSD = 100 cm.

The results (Fig. 3) show that pretreatment with sodium azide or hydroxylamine had a protective action. The result with azide is similar to that obtained by Bacq (1951), but the result with hydroxylamine appears to be a new finding in general agreement with Bacq's work.

FIG. 3.—Survival of mice after X-irradiation.

1 : Control. 2 : Injected with 1·5 mg. per kg. $NaN_3$ 15 min. before irradiation. 3 : Injected with 15 mg. per kg. $NaN_3$ 15 min. after irradiation. 4 : Injected with 25 mg. per kg. hydroxylamine hydrochloride 15 min. before irradiation. 5 : Injected with 15 mg. per. kg. $NaN_3$ 15 min. before irradiation.

### DISCUSSION.

The attempt to increase sensitivity of tumours to X-rays by poisoning the catalase of grafted tumours *in vivo* with azide or hydroxylamine was unsuccessful. This might be because the amounts of hydrogen peroxide produced in tumour metabolism are too small to have any effect in augmenting the action of radiation. The hydrogen peroxide may, however, be produced and utilised in the tumour tissue. The absence of increased sensitivity is, however, more probably due to the fact that the catalase poisons used are also agents which protect animals from radiation. These substances may combine or neutralise free hydroxyl radicals. For these reasons work should be carried out to measure tumour peroxidases and try to inhibit these as well as catalase, and to find catalase poisons which are not protective agents against radiation.

The search for a poison more specific for catalase than is azide was also unsuccessful. Sodium azide and hydroxylamine are much the most specific catalase poisons of the compounds examined. *p*-Hydroxyphenylazide had only 1/50 of the activity of sodium azide (calculated on a molar basis). This difference is exactly the same as that found between hydroxylamine and phenylhydroxylamine. On the other hand, phenylhydrazine appears to be a more potent inhibitor than hydrazine (Blaschko, 1935*a*). The introduction of a methyl group into the hydroxylamine molecule (o-methyl-hydroxylamine) reduced the activity 400 fold. The inhibition produced by diethyl-*p*-phenylenediamine was of the same order as that described for dimethyl-*p*-phenylenediamine by Horner and Betzel (1950).

The figures on the ratio of catalase inhibiting concentration to lethal dose show that sodium azide is the most favourable catalase poison as well as the most active. The median lethal dose of sodium azide should give a concentration of azide which is one thousand times that causing 50 per cent inhibition of catalase. Thus, sodium azide is probably the safest catalase poison for use *in vivo* in spite of its high toxicity.

The increase in poisoning activity of azide with decrease in the pH is similar to that found for the effect of formate and acetate by Agner and Theorell (1946), who showed that anions in general combine with catalase and inactivate it to an extent increasing with decrease in pH. These authors explained this as due to the anion displacing the hydroxyl group of the catalase and it is possible that azide acts in the same way. The data of Agner and Theorell (1946) on poisoning of catalase with formate at different pH values are analogous to those found for azide (Table IV) as shown in Table XI. The calculated concentration of undissociated formic acid which causes 50 per cent inhibition is seen to be constant for any one temperature and so analogous to our results with azide.

TABLE XI.—*Inhibition of Catalase by Formate at Different pH Values (data from Agner and Theorell* 1946).

| pH. | Temp. | Conc. formate for 50% inhibition. | Proportion undissociated (%). | Calculated conc. of undissociated formic acid. |
|---|---|---|---|---|
| 3·37 | 0·8° | $2 \times 10^{-6}$ | 75 | $1·5 \times 10^{-6}$ |
| 5·18 | 0·8° | $6 \times 10^{-5}$ | 2 | $1·2 \times 10^{-6}$ |
| 3·35 | 20° | $5 \times 10^{-6}$ | 75 | $3·7 \times 10^{-6}$ |
| 3·99 | 20° | $10^{-5}$ | 40 | $4 \times 10^{-6}$ |
| 5·20 | 20° | $2 \times 10^{-4}$ | 2 | $4 \times 10^{-6}$ |

These findings may be interpreted as Agner and Theorell (1946) suggest as due to replacement of the OH of the catalase by azide or formate. The fact that the inhibition seems to depend on the concentration of unionised poison suggests that it is the undissociated molecule which combines with the enzyme. Now these effective poisons resemble the substrate in shape. It is established that the atoms of hydrazoic acid are in a chain rather than in a ring, and some other simple catalase poisons are of about the same size and shape as hydrogen peroxide (Table XII). In hydroxylamine one of the oxygen atoms is replaced by an NH group, and in hydrazine both oxygen atoms are so replaced by NH

TABLE XII.—*The Substrate and Poisons of Catalase.*

| | | | | |
|---|---|---|---|---|
| Hydrogen peroxide | H | O | O | H |
| Hydrazoic acid | H | N | N | N |
| Hydroxylamine | H | O | NH | H |
| o-Methylhydroxylamine | $CH_3$ | O | NH | H |
| Hydrazine | H | NH | NH | H |
| Methylamine | H | $CH_2$ | NH | H |
| Nitrous acid | H | O | N | O |
| Formic acid | H | O | CO | H |
| Acetic acid | H | O | CO | $CH_3$ |

groups. In formic acid one oxygen atom of hydrogen peroxide is replaced by a CO group. The replacement of a hydrogen of formic acid by $CH_3$ as in acetic acid reduces the activity 800 fold (Agner and Theorell, 1946), which is the same order as that produced by the introduction of an o-methyl group in hydroxylamine.

Thus the poisons may combine readily with catalase, possibly replacing a hydroxyl group or hydrogen peroxide because of their shape and size. In testing this hypothesis, methylamine was found to be a poison (of the same order as hydrazine), but ethyl formate and formamide, which it was hoped would be as effective as undissociated formic acid, were both found to be inactive.

While the poisoning with hydrazoic acid and formic acid may depend upon the entire undissociated molecule, the poisoning with hydroxylamine and hydrazine appeared to be independent of the pH. Now in the cases of hydrazoic acid and formic acid, ionisation would result in loss of a hydrogen atom (and gain of a charge), so that the ion would have less structural resemblance to hydrogen peroxide. On the other hand, the salts of hydroxylamine and hydrazine have added hydrogen atoms and charge, which presumably makes little difference to the poisoning action. These results show that although the shape of the molecule may be important, it is not the only factor controlling catalase inhibition for this type of poison.

Catalase poisons may be divided into at least three groups :

(a) Those combining with iron, such as cyanide and sulphide.

(b) Those combining with the enzyme possibly because of a formal resemblance to the substrate such as azide and hydroxylamine.

(*c*) Those which act as substrates for peroxidase such as *p*-phenylenediamine and hydroquinone (Stern and Bird, 1951).

### SUMMARY.

(1) A list of catalase poisons including some hitherto unknown poisons is given, and the concentration required for 50 per cent inhibition of catalase is compared with the lethal dose for mice. Azide and hydroxylamine are the most specific of known catalase poisons.

(2) The poisoning with azide increases with decrease in pH so that the concentration of undissociated hydrazoic acid required for 50 per cent inhibition remains constant. The poisoning by hydroxylamine and hydrazine is almost independent of pH. The poisoning of catalase is considered in relation to these findings that hydrazoic acid and formic acid act as undissociated molecules. The possibility that poisons of this type are effective because of their structural resemblance to hydrogen peroxide is discussed.

(3) Poisoning of catalase of liver and tumour of rats was demonstrated after injection of azide.

(4) Injection of small doses of azide or hydroxylamine into rats immediately before X-irradiation did not increase the radiosensitivity of tumours. Injection of large doses of azide or hydroxylamine into mice before irradiation reduced the mortality from X-irradiation of the whole body.

(5) Catalase activity could be expressed as a $Q_{O_2}^{1.29 \times 10^{-5}\ H_2O_2}$ value in $\mu$l. $O_2$ which would be liberated, per hour, per mg. dry weight of tissue; this has the same numerical value as the Kat.f.

Since this paper was sent for publication the authors have seen the communications of B. Chance (1952) in which the poisoning of catalase by azide and formate is shown to depend on the concentration of undissociated molecules of the acids concerned.

One of us (E. G.) wishes to thank the British Council for a scholarship held while the work was carried out. We are indebted to Miss E. B. Harriss, Miss M. Winsborough and Dr. L. F. Lamerton of the Physics Department of the Royal Cancer Hospital for help in the irradiation experiments, and to Mrs. S. Sargent, M.Sc., for the toxicity data of Table II. The investigation was supported by grants to the Royal Cancer Hospital and Chester Beatty Research Institute from the British Empire Cancer Campaign, the Jane Coffin Childs Memorial Fund for Medical Research, the Anna Fuller Fund, and the National Cancer Institute of the National Institutes of Health, U.S. Public Health Service.

### REFERENCES.

AGNER, K., AND THEORELL, H.—(1946) *Arch. Biochem.*, **10**, 321.

BACQ, Z. M.—(1951) *Experientia*, **8**, 11.

BLASCHKO, H.—(1935*a*) *Biochem. J.*, **29**, 2303.—(1935*b*) *J. Physiol.*, **84**, 52P.

BUTLER, J. A. V., AND SMITH, K. A.—(1950) *J. chem. Soc.*, 3411.

CHANCE, B.—(1952) *J. Biol. Chem.* **194**, 471, 483.

CONWAY, B., AND BUTLER, J. A. V.—(1952) *Ibid.*, in press.

CRABTREE, H. G., AND CRAMER, W.—(1933) *Proc. Roy. Soc.*, **B. 113**, 226.

EULER, H. VON, AND GLASER, A.—(1950) *Dtsch. med. Wschr.*, **75**, 631.

*Idem* AND JOSEPHSON, K.—(1927) *Leibigs Ann.*, 455, 1.

FOULKES, E. C., AND LEMBERG, R.—(1949) *Enzomologia*, **13**, 302.

HERBERT, D., AND PINSENT, J.—(1948) *Biochem. J.*, **43**, 193.

HOLLINGER, N., FUHRMAN, F. A., LEWIS, J. J., AND FIELD, J.—(1949) *J. cell. comp. Physiol.*, **33**, 223.

HORNER, L., AND BETZEL, C.—(1950) *Leibigs Ann.*, **571**, 225.

KEILIN, D.—(1936) *Proc. Roy. Soc.*, **B. 121**, 165.

*Idem* AND HARTREE, E. F.—(1934) *Nature*, **134**, 933.

RONA, P., FIEGEL, A., AND NAKAHARA, W.—(1925) *Biochem. J.*, **160**, 272.

SCHOLER, W., AND MEIER, R.—(1944) *Helv. physiol. pharmacol. Acta*, **2**, 83.

SEIDE, G.—(1941) *Biochem. Z.*, **308**, 175.

STERN, K. G.—(1932) *Z. physiol. Chem.*, **209**, 176.

STERN, R., AND BIRD, L. H.—(1951) *Biochem. J.*, **49**, 335.

TAYLOR, B., GREENSTEIN, J. P., AND HOLLAENDER, A.—(1947) *Cold Spring Harbor Symposia*, **12**, 237.